# Synthesis of RpoS Is Dependent on a Putative Enhancer Binding Protein Rrp2 in *Borrelia burgdorferi*

**DOI:** 10.1371/journal.pone.0096917

**Published:** 2014-05-08

**Authors:** Zhiming Ouyang, Jianli Zhou, Michael V. Norgard

**Affiliations:** Department of Microbiology, University of Texas Southwestern Medical Center, Dallas, Texas, United States of America; Umeå University, Sweden

## Abstract

The RpoN-RpoS regulatory pathway plays a central role in governing adaptive changes by *B. burgdorferi* when the pathogen shuttles between its tick vector and mammalian hosts. In general, transcriptional activation of bacterial RpoN (σ^54^)-dependent genes requires an enhancer binding protein. *B. burgdorferi* encodes the putative enhancer binding protein Rrp2. Previous studies have revealed that the expression of σ^54^-dependent *rpoS* was abolished in an *rrp2* point mutant. However, direct evidence linking the production of Rrp2 in *B. burgdorferi* and the expression of *rpoS* has been lacking, primarily due to the inability to inactivate *rrp2* via deletion or insertion mutagenesis. Herein we introduced a regulatable (IPTG-inducible) *rrp2* expression shuttle plasmid into *B. burgdorferi*, and found that the controlled up-regulation of Rrp2 resulted in the induction of σ^54^-dependent *rpoS* expression. Moreover, we created an *rrp2* conditional lethal mutant in virulent *B. burgdorferi*. By exploiting this conditional mutant, we were able to experimentally manipulate the temporal level of Rrp2 expression in *B. burgdorferi*, and examine its direct impact on activation of the RpoN-RpoS regulatory pathway. Our data revealed that the synthesis of RpoS was coincident with the IPTG-induced Rrp2 levels in *B. burgdorferi*. In addition, the synthesis of OspC, a lipoprotein required by *B. burgdorferi* to establish mammalian infection, was rescued in the *rrp2* point mutant when RpoS production was restored, suggesting that Rrp2 influences *ospC* expression indirectly via its control over RpoS. These data demonstrate that Rrp2 is required for the synthesis of RpoS, presumably via its action as an enhancer binding protein for the activation of RpoN and subsequent transcription of *rpoS* in *B. burgdorferi.*

## Introduction

*Borrelia burgdorferi*, the causative agent of Lyme disease, is sustained in nature via a complex life cycle involving an arthropod tick vector (*Ixodes scapularis*) and mammals [1], [2]. During its transit between these two markedly different host and tick milieus, *B. burgdorferi* undergoes significant adaptive changes. In *B. burgdorferi*, host adaptation is achieved by dramatic changes in gene expression in response to various tick or host stimuli [3]–[9]. Among a number of potential regulators that have been postulated to be present in *B. burgdorferi* [10]–[45], a novel genetic regulatory pathway, the RpoN-RpoS pathway (or the σ^54^-σ^S^ cascade) [18], plays a central role in modulating *B. burgdorferi* host adaptive responses and virulence expression. In this pathway, one alternative sigma factor (σ^54^, RpoN) controls the expression of another alternative sigma factor (σ^S^, RpoS) through binding to a canonical −24/−12 promoter sequence [12], [26], [40]. Once RpoS is produced, it functions as a master regulator to modulate the expression of a number of virulence-associated outer membrane lipoproteins such as outer surface lipoproteins (Osp) C and A, and decorin binding proteins (Dbp) B and A [4], [8], [12]–[14], [18], [29], [32], [36], [40], [46]–[57].

Transcriptional activation of σ^54^-dependent genes in bacteria requires a bacterial enhancer binding protein (bEBP), which is an AAA+ activator ATPase [58]–[63]. Sequence analyses have indicated that Rrp2 (BB0763) is composed of three structural domains, including an N-terminal regulatory domain (R), a central AAA+ ATPase core domain (C), and a C-terminal DNA binding domain (D), suggesting that Rrp2 may function as a bEBP to activate σ^54^–dependent *rpoS* transcription in *B. burgdorferi* [15], [45]. Previously, by exploiting a variant carrying a point mutation G239C in the C domain of Rrp2, we [10], [29], [45] reported that the *rrp2* point mutant was incapable of expressing *rpoS* and virulence-associated factors such as OspC and DbpA, suggesting that, as expected, Rrp2 is essential for activation of the RpoN-RpoS regulatory pathway. Despite this important finding, there remain many unanswered questions concerning the roles of Rrp2 in *B. burgdorferi* gene regulation. In particular, the finding that expression of *rpoS* was abolished in the *rrp2* point mutant has been hitherto the only evidence to support the role of Rrp2 in the activation of the RpoN-RpoS pathway. A direct link between Rrp2 protein levels produced in *B. burgdorferi* and the expression of *rpoS* has been lacking, primarily due to the inability to inactivate *rrp2* via deletion or insertion mutagenesis. The G239C point mutation in *rrp2* presumably abolishes the putative ATPase activity required for σ^54^–dependent *rpoS* transcriptional activation. However, it also remains possible that the G239C point mutation causes a change in Rrp2’s overall conformation, thereby preventing *rpoS* transcription in the *rrp2* point mutant. In addition, although the expression of *ospC* was found to be lost in the *rrp2* point mutant, how Rrp2 ultimately controls the expression of this key virulence-associated lipoprotein remains unknown. Rrp2 may indirectly modulate *ospC* expression via its control over RpoS. Alternatively, given that *ospC* was found to be constitutively expressed in *B. burgdorferi* when *ospAB* and *rpoS* were inactivated [64], Rrp2 may control *ospC* expression through another RpoS-independent factor(s). It is also possible that Rrp2 may directly modulate *ospC* expression by binding to its promoter. To address these questions, we employed an artificial gene expression system [65], [66] to experimentally control the protein levels of Rrp2 synthesized in *B. burgdorferi*, and examined its impact on *rpoS* expression. Such a strategy affects only the levels of Rrp2 produced in *B. burgdorferi*, and does not alter the overall structure of the protein. Our data show that the expression level of Rrp2 correlates closely with the expression of *rpoS*, indicating that Rrp2 activates the expression of σ^54^–dependent *rpoS* which, in turn, modulates *ospC* expression in *B. burgdorferi*.

## Materials and Methods

### Bacterial Strains and Culture Conditions

All strains and plasmids used in this study are described in Table 1. Low-passage infectious wild-type *B. burgdorferi* strain 297 [67], and the *rrp2* point mutant OY01 [29], were routinely cultured at 37°C and 5% CO_2_ in either BSK-II medium [68] or BSK-H medium (Sigma) supplemented with 6% rabbit serum (Pel-Freeze). When appropriate, supplements were added to media at the following concentrations: erythromycin, 60 ng/ml; kanamycin, 150 µg/ml; streptomycin, 100 µg/ml. Spirochetes were enumerated by dark-field microscopy. *E. coli* strains were cultured in Lysogeny Broth (LB) supplemented with appropriate antibiotics at the following concentrations: ampicillin, 100 µg/ml; kanamycin, 50 µg/ml; spectinomycin, 100 µg/ml.

**Table 1:** Strains and plasmids used in this study.

Strains or plasmids	Description	Source
***B. burgdorferi***		
297	Infectious *B. burgdorferi*, human spinal fluid isolate	[67]
OY01	297, *rrp2*(G239C) point mutant	[29]
AH206	297, *rpoS*::ermC	[18]
OY159	297 transformed with pRrp2	This study
OY160	OY01 transformed with pRrp2	This study
OY173	OY01 transformed with pRrp2-FLAG	This study
OY179	*rrp2* conditional lethal mutant, OY173 transformed with pOY202	This study
***E. coli*** Top10F′	F′[*lacI* ^q^ Tn*10*(Tet^r^)] *mcrA* Δ(*mrr-hsdRMS-mcrBC*) φ80*lacZ*ΔM15ΔlacX74 recA1 araΔ*139* Δ (ara-leu)*7697 galU galK rpsL*(Str^r^) *endA1 nupG*	Invitrogen
**Plasmids**		
pGEM-Teasy	TA cloning vector; Amp^r^	Promega
pJSB275	Shuttle vector, Spec/Strep^r^	[69]
pRpoS	IPTG-inducible *rpoS* expression construct	[30]
pOY100	PCR product of 86F/87R cloned into pGEM-Teasy, amp^r^	This study
pJD55	Shuttle vector, Spec/Strep^r^	[46]
pOY202	PflgB-kan cloned into pOY100, Amp/Kan^r^	This study
pRrp2	PCR product of 303F/303R cloned into pJSB275, Spec/Strep^r^	This study
pRrp2-FLAG	PCR product of 303F/263R cloned into pJSB275, Spec/Strep^r^	This study

### Generation of the IPTG-inducible *rrp2* Expression Construct

To experimentally control *rrp2* expression in *B. burgdorferi*, one *rrp2* expression construct pRrp2 was generated by using the *lac*-based gene inducible expression system [65], [66]. Briefly, *rrp2* was amplified from *B. burgdorferi* using primers ZM303F and 303R (Table S1), and then cloned into pJSB275 [69] at the NdeI site. In pRrp2, *rrp2* transcription is directly controlled by the IPTG-inducible T5 promoter (a.k.a. the promoter PpQE30) from plasmid pQE30 (Qiagen).

### Generation of the *rrp2* Conditional Lethal Mutant OY179

To create an *rrp2* conditional mutant, we first generated one shuttle plasmid pRrp2-FLAG in which *rrp2* expression was placed under the control of the IPTG-inducible promoter PpQE30. For creating pRrp2-FLAG, a DNA fragment encoding Rrp2-FLAG was amplified by using ZM303F and ZM263R (Table S1), and cloned into pJSB275 [69] at the NdeI site. This strategy adds a FLAG-tag (DYKDDDDK) to the C-terminus of Rrp2, thereby facilitating the detection of Rrp2 in *B. burgdorferi*. The plasmid pRrp2-FLAG was then electroporated into the *rrp2* point mutant OY01, yielding the streptomycin-resistant strain OY173. *B. burgdorferi* transformation was performed as previously described [29], [70].

To inactivate *rrp2* in *B. burgdorferi* through homologous recombination, a suicide plasmid pOY202 was created. Briefly, the left arm for creating pOY202 was PCR-amplified using primers ZM86F and ZM86R, whereas the right arm was amplified using ZM87F and ZM87R (Table S1). After digestion with AscI, these two fragments were fused together. By using this ligated DNA as the template, PCR was employed to amplify a fragment comprising the upstream and downstream regions of *rrp2* by using primers ZM86F and ZM87R. The obtained fragment was cloned into pGEM-Teasy vector (Promega), yielding pOY100. The P*flgB-*Kan cassette, excised from pJD55 [46] using AscI, was cloned into pOY100 at the AscI site. In the resulting construct pOY202, the P*flgB-*Kan cassette was inserted in *rrp2* in the opposite direction as transcription of *csrA*. All constructs were confirmed using PCR amplification, restriction digestion, and sequence analysis. The plasmid pOY202 was then transformed into *B. burgdorferi* strain OY173. Transformants were isolated in the presence of kanamycin and streptomycin, along with 0.05 mM of IPTG to allow the production of Rrp2-FLAG during selection.

### RNA Isolation and qRT-PCR

RNA isolation and qRT-PCR were performed as previously described [29]–[31], [71]. Briefly, when total RNA was isolated from *B. burgdorferi* by using Trizol (Invitrogen), RNase-free DNase I (GenHunter Corporation) was used to digest genomic DNA. After RNA was further purified using RNeasy Mini Kit (Qiagen), 1 µg of RNA was used to synthesize cDNA using the SuperScript III Platinum Two-step qRT-PCR kit according to the manufacturer’s protocol (Invitrogen). qRT-PCR was employed to examine gene expression, using the relative quantification method (ΔΔ*C*T) as described [29]–[31], [71]. Gene expression fold change was presented as mean ± SE values from three independent experiments. Statistical analyses of the data were performed using the Student’s *t* test.

### SDS-PAGE and Semi-quantitative Immunoblot Analyses

A volume of whole cell lysate equivalent to 4×10^7^ spirochetes was loaded per lane onto a 12.5% acrylamide gel. Resolved proteins were either stained with Coomassie brilliant blue or transferred to nitrocellulose membrane for immunoblot analysis as previously described [29]–[31], [71]. Rrp2, FLAG, RpoS, OspC, and DbpA were detected using anti-Rrp2 monoclonal antibody 5B8-100-A1, anti-FLAG M2 monoclonal antibody (Sigma), anti-RpoS monoclonal antibody 6A7-101, anti-OspC monoclonal antibody 1B2-105A, or anti-DbpA monoclonal antibody 6B3, respectively. Immunoblots were developed colorimetrically using 4-chloro-1-napthol as the substrate or by chemiluminescence using the ECL Plus Western Blotting Detection system (Amersham Biosciences). Images were documented by using a Fujifilm LAS-3000 Imager (Fujifilm), and semi-quantitative analyses were performed by using the MultiGauge V3.0 software (Fujifilm).

## Results and Discussion

### Complementation of the *rrp2* Point Mutation by using an IPTG-inducible *rrp2* Expression Construct

**Figure 1:**
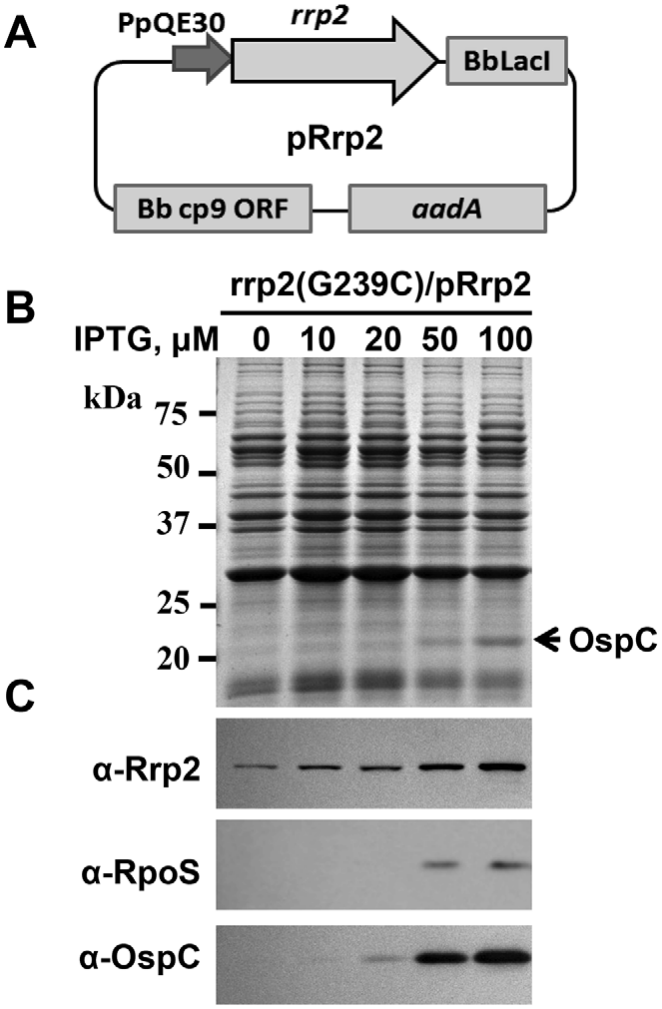
*Trans-*complementation of an *rrp2* point mutant using a shuttle plasmid. (A) Construction of an IPTG-inducible *rrp2* expression shuttle plasmid. To create pRrp2, *rrp2* was amplified from *B. burgdorferi* and cloned into pJSB275. The plasmid pRrp2 was then introduced into strain OY01 (*rrp2*[G239C]), yielding OY160. SDS-PAGE (B) and immunoblot (C) were performed to analyze gene expression in OY160. Bacteria were grown at 37°C in BSK-II medium with various concentrations of IPTG. When bacterial growth reached ∼10^8^ cells per ml, spirochetes were harvested. Approximately 4×10^7^ spirochetes were loaded onto each lane of a 12.5% SDS-PAGE gel. In (B), approximate molecular masses are indicated at the left in kDa; concentrations of IPTG are indicated above the image; and the arrow indicates OspC. Specific antibodies, denoted as α- used in the immunoblot (C), are indicated on the left.

Previously, we introduced a G239C mutation into *rrp2* and created an *rrp2* point mutant OY01 (*rrp2*[G239C]) in *B. burgdorferi* [29]. The expression of *rpoS* and *ospC* is abolished in OY01. To confirm that the loss of *rpoS* and *ospC* expression in the *rrp2* point mutant was due solely to the mutation of *rrp2*, a *trans*-complementation approach was employed. To this end, an IPTG-inducible plasmid pRrp2 (Fig. 1A) was created by placing the expression of *rrp2* under the control of the IPTG-inducible PpQE30 promoter. By adjusting the amount of the inducer (IPTG) added to the medium, the expression of *rrp2* on pRrp2 could be controlled. pRrp2 was then introduced into the *rrp2* point mutant OY01, yielding the streptomycin-resistant strain OY160.

To induce the expression of wild-type Rrp2, OY160 was grown in BSK-II medium containing varying concentrations of IPTG. In this experiment and subsequently, bacterial growth and morphology were not affected when spirochetes were grown in media with indicated levels of IPTG. Cells were collected when bacterial growth reached early stationary phase (∼10^8^ cells/ml). As shown in Fig. 1C, when 10-, 20-, 50-, or 100-µM of IPTG was added into the media, synthesis of Rrp2 was enhanced in a dose-dependent manner in *B. burgdorferi*. Of note, when IPTG was not added into the medium, a band was also detected in immunoblot by using the Rrp2 antibody; this band represents the existing mutated protein Rrp2(G239C). The induction of Rrp2 also resulted in the synthesis of RpoS and OspC in OY160. As shown in Fig. 1B and Fig. 1C, when bacteria were grown in BSK-II containing 50- or 100-µM of IPTG, RpoS and OspC were readily detected in OY160, and the increased protein levels correlated well with the increased levels of Rrp2 in OY160. These data suggest that Rrp2 expressed from pRrp2 is capable of complementing the *rrp2*(G239C) point mutation, thereby activating the RpoN-RpoS pathway in *B. burgdorferi*.

### Up-regulation of Rrp2 in *B. Burgdorferi* Induces the Expression of *RpoS* and *OspC*

**Figure 2:**
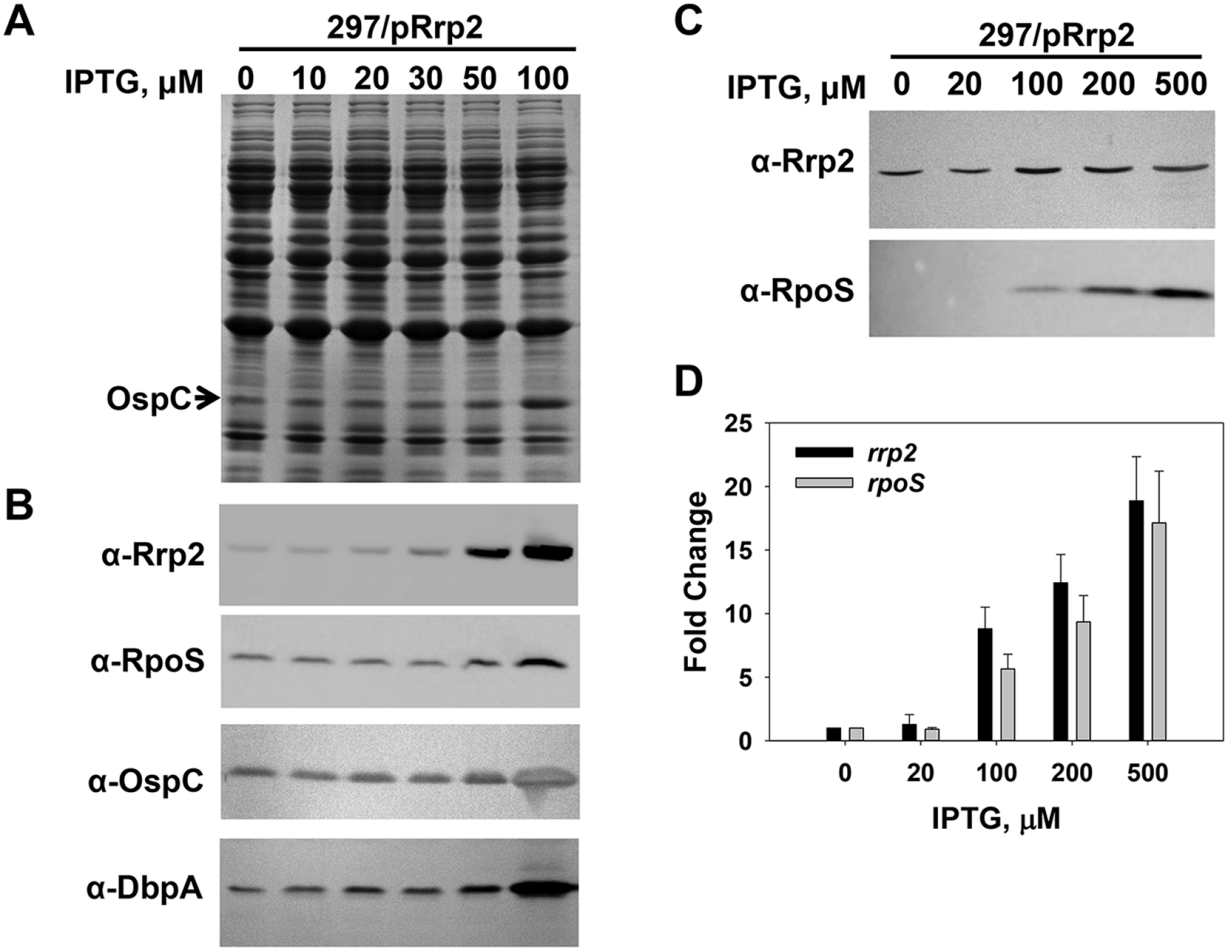
Up-regulation of Rrp2 in *B. burgdorferi* induces the expression of *rpoS*. Gene expression in OY159 was analyzed by SDS-PAGE (A), immunoblot (B, C), and qRT-PCR analyses (D). In (A) and (B), spirochetes grown in BSK-II media containing varying concentrations of IPTG were harvested when bacterial growth reached early stationary phase (∼10^8^ cells per ml). In (C) and (D), spirochetes were grown in BSK-II medium. When bacterial growth reached mid-log phase (∼10^7^ cells per ml), various amounts of IPTG were added into culture. Cells were collected at 9 h post-induction. In (A) and (C), concentrations of IPTG are indicated above the image. The arrow indicates OspC in (A). Specific antibodies, denoted as α- used in the immunoblot (B, C), are indicated on the left. In (D), data were collected from three independent experiments, and the bars represent the mean measurements ± standard deviation. The mean values between induced groups (100-, 200-, or 500 µM IPTG) and the uninduced group (0 µM IPTG) were compared using the Student’s *t* test and are significantly different (p<0.05). For data normalization, the *B. burgdorferi flaB* gene was used as an internal control.

The approach of gene overexpression has proven to be a highly valuable tool for examining gene functions, particularly for genes that cannot be inactivated [72]. This strategy was successfully employed previously to study the role of Rrp2 in *B. burgdorferi*, where it was reported that overexpression of Rrp2 in *B. burgdorferi* led to the induction of OspC [69]. However, it remained unknown whether RpoS synthesis was influenced by the overexpression of Rrp2 in *B. burgdorferi*. In this current study, the IPTG-inducible *rrp2* expression construct pRrp2 was introduced into *B. burgdorferi* wild-type strain 297, yielding the merodiploid strain OY159. Gene expression in these spirochetes was measured through SDS-PAGE, semi-quantitative immunoblot, and quantitative RT-PCR analyses. In this experiment, two strategies employing shorter or longer induction times were used to induce Rrp2 in *B. burgdorferi*. For the longer induction time, bacteria were continuously grown in BSK-II containing various amounts of IPTG for about 7 days and harvested when growth reached the early stationary phase (∼10^8^ cells/ml). As shown in Fig. 2B, synthesis of Rrp2 in OY159 was enhanced when bacteria were grown in media containing IPTG (compared with spirochetes cultivated in media without IPTG). Moreover, synthesis of RpoS and OspC was also found to be enhanced when IPTG was added into the medium (Fig. 2A, 2B). To exclude the possibility that gene induction might be an indirect effect resulting from prolonged exposure to IPTG, a shorter (9 h) induction period was also examined. When bacterial growth in BSK-II reached mid-log phase (∼10^7^ cells/ml), varying amounts of IPTG were added into the media. After 9 h of induction, spirochetes were collected and gene expression was examined. As shown in Fig. 2C, immunoblot analyses showed that the syntheses of both Rrp2 and RpoS were induced by IPTG in a dose-dependent manner. Gene expression was also measured by using real-time quantitative RT-PCR (qRT-PCR) analyses. When gene transcription in spirochetes grown in BSK-II with 20-, 100-, 200-, or 500-µM of IPTG was compared with gene expression in bacteria grown without IPTG, transcription of *rrp2* was induced at 1.3-, 8.8-, 12.4-, or 18.9-fold, respectively; accordingly, *rpoS* transcription was induced at 0.9-, 5.6-, 9.4-, or 17.1-fold, respectively. These combined data strongly suggest that Rrp2 is responsible for the induction of RpoS in *B. burgdorferi*.

### Generation of an *rrp2* Conditional Mutant in *B. burgdorferi*

**Figure 3:**
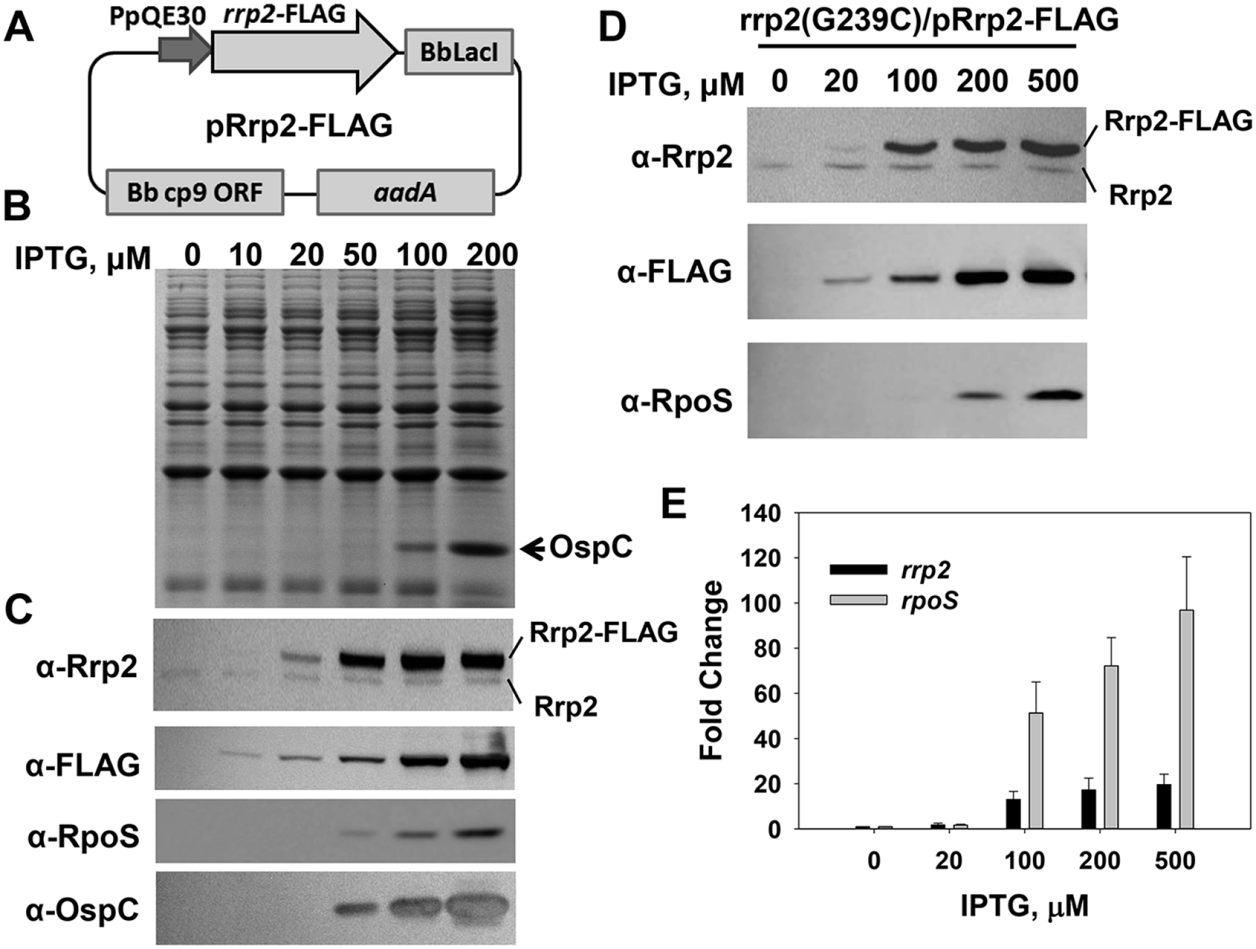
Gene expression in *B. burgdorferi* strain OY173. (A) Construction of an IPTG-inducible *rrp2*-FLAG expression shuttle plasmid. The plasmid pRrp2-FLAG pRrp2 was introduced into strain OY01 (*rrp2*[G239C]), yielding OY173. SDS-PAGE (B), immunoblot (C, D), and qRT-PCR analyses (E) were performed to analyze gene expression. In (B) and (C), spirochetes grown in BSK-II medium containing varying concentrations of IPTG were harvested when bacterial growth reached early stationary phase (∼10^8^ cells per ml). In (D) and (E), spirochetes were grown in BSK-II medium. When bacterial growth reached mid-log phase (∼10^7^ cells per ml), various amounts of IPTG were added into culture. Cells were collected at 9 h post-induction. In (B) and (D), concentrations of IPTG are indicated above the image. The arrow indicates OspC in (B). Specific antibodies, denoted as α- used in the immunoblot (C, D), are indicated on the left. In (E), the bars represent the mean measurements ± standard deviation. The mean values between induced groups (100-, 200-, or 500 µM IPTG) and the uninduced group (0 µM IPTG) were compared using the Student’s *t* test and are significantly different (p<0.05). For data normalization, the *B. burgdorferi flaB* gene was used as an internal control.

Given that many attempts to fully inactivate *rrp2* in *B. burgdorferi* have failed, and that *rrp2* thus seems to be essential for *B. burgdorferi in vitro* growth [69], we generated a *rrp2* conditional mutant in *B. burgdorferi* using a similar approach as described previously [69], [73]. In this conditional lethal mutant, the wild-type chromosomal copy of *rrp2* is disrupted; *rrp2* is expressed from an IPTG-inducible shuttle plasmid. As a prelude to this approach, we first created another IPTG-inducible *rrp2* expression construct pRrp2-FLAG (Fig. 3A). From this shuttle plasmid, the expression of *rrp2* in *B. burgdorferi* is tightly controlled by IPTG added into the medium. To assist in the detection of Rrp2, a DNA fragment encoding the FLAG tag was fused to the 3′ of *rrp2*. Because adding a FLAG tag to Rrp2 could affect its general function, this plasmid pRrp2-FLAG was initially introduced into the *rrp2* point mutant OY01 (*rrp2*[G239C]) to create the strain OY173. OY173 was then used to test whether Rrp2-FLAG was functional in activating σ^54^-dependent *rpoS* expression. OY173 bacteria were grown continuously in media with varying concentrations of IPTG, and collected when growth reached the early stationary phase. As shown in Fig. 3C, when IPTG was not added into the medium, Rrp2-FLAG was not detected. However, when 20-, 50-, 100-, or 200-µM of IPTG was added into the media, the synthesis of Rrp2-FLAG was enhanced in a dose-dependent manner. The production of Rrp2-FLAG also resulted in the synthesis of RpoS and OspC in OY173. As shown in Fig. 3B and 3C, when bacteria were grown in BSK-II containing 50-, 100-, or 200-µM of IPTG, RpoS and OspC were readily detected in OY173, and the protein levels correlated closely with the increased levels of Rrp2-FLAG produced in *B. burgdorferi*. A 9-hr induction experiment was also carried out to examine gene induction in OY173. As shown in Fig. 3D, when bacteria at mid-log phase were exposed to 20-, 100-, 200-, or 500-µM of IPTG for 9 h, the synthesis of Rrp2-FLAG was readily detected in OY173. RpoS was also efficiently expressed in this strain (Fig. 3D). Moreover, qRT-PCR analyses revealed that exposing bacteria to IPTG for 9 h resulted in the concomitant induction of *rrp2* and *rpoS* (Fig. 3E). Taken together, these data suggested that Rrp2-FLAG expressed from pRrp2-FLAG was capable of functioning like native Rrp2 to activate the expression of σ^54^-dependent *rpoS*.

**Figure 4:**
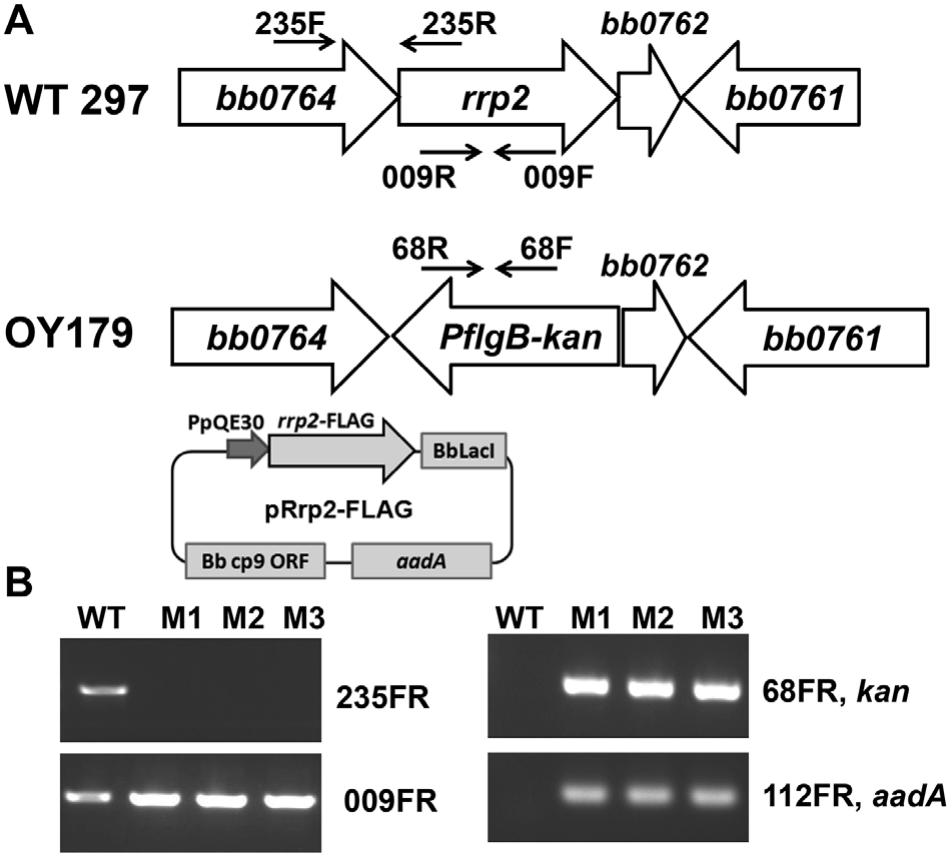
Generation of an *rrp2* conditional lethal mutant in *B. burgdorferi*. (A) Schematic representation of the *bb0764*–*bb0761* genes in the *B. burgdorferi* chromosome and the insertion of PflgB-kan cassette into *rrp2* by homologous recombination. Arrows indicate the approximate positions of the oligonucleotide primers used for subsequent analyses. (B) Analyses of the wild-type 297 and the *rrp2* conditional lethal mutant OY179 by PCR. The specific primer pairs are indicated on the right. Lanes WT, 297; lanes M1, M2, and M3, three clones of OY179.

Next, to fully inactivate *rrp2*, a suicide vector pOY202 that would target *rrp2* was first introduced into strain 297, but no transformants could be recovered. We then introduced pOY202 into strain OY173 (containing pRrp2-FLAG), and the desired transformants were selected by using streptomycin and kanamycin, together with IPTG (IPTG was used to induce *rrp2* expression from pRrp2-FLAG). Through allelic exchange, the chromosomal copy of *rrp2* was replaced by the PflgB-kan cassette, yielding strain OY179 (Fig. 4A). The inactivation of chromosomal *rrp2* was confirmed by PCR analyses. As shown in Fig. 4B, by using primers ZM235F and ZM235R, a 941-bp fragment spanning *bb0764* and *rrp2* was successfully amplified from WT strain 297 (lane 1), but not from the conditional mutant OY179 (lanes M1, M2, and M3). Also, PCR amplification revealed that strain OY179 contained the *kan* gene (conferring kanamycin resistance) and the *aadA* gene (conferring streptomycin resistance) (Fig. 4B).

The growth of the *rrp2* conditional mutant OY179 was assessed by cultivating the bacteria in BSK-II medium containing various concentrations of IPTG. OY179 grew in BSK-II with 20-, 30-, 50-, or 100-µM IPTG, but failed to grow in BSK-II without IPTG (Fig. S1). Consistent with previous observations [69], we also conclude that *rrp2* is essential for *B. burgdorferi* growth *in vitro*.

### Expression of *rrp2* Correlates with the Expression of *RpoS* and *OspC* in the *rrp2* Conditional Mutant

**Figure 5:**
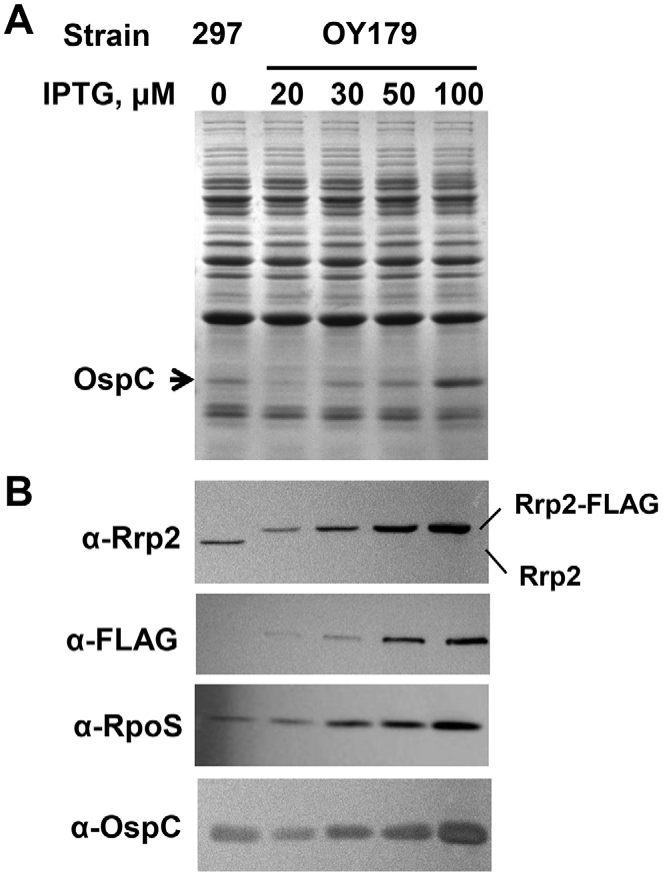
Gene expression in the *rrp2* conditional lethal mutant OY179. SDS-PAGE (A) and semi-quantitative immunoblot (B) analyses were performed to analyze gene expression. Bacteria were grown at 37°C in BSK-II medium with various concentrations of IPTG. When bacterial growth reached ∼10^8^ cells per ml, spirochetes were harvested. Approximately 4×10^7^ spirochetes were loaded onto each lane of a 12.5% SDS-PAGE gel. Concentrations of IPTG are indicated above the image, and the arrow in (A) indicates OspC. Specific antibodies, denoted as α- used in the immunoblot (B), are indicated on the left.

To assess whether the protein level of Rrp2 correlated with the expression of *rpoS*, gene expression in OY179 was measured via SDS-PAGE and semi-quantitative immunoblot analyses. To this end, OY179 was grown in BSK-II with varying concentrations of IPTG and cultures were harvested when cell growth reached early stationary phase. For bacteria grown under these conditions, no obvious differences were observed when spirochete morphology and motility were examined using dark-field microscopy. As shown in Fig. 5B, immunoblot analyses revealed that OY179 produced Rrp2-FLAG but not native Rrp2. Moreover, the levels of Rrp2-FLAG were dependent on the concentrations of the inducer IPTG. Specifically, when 20-µM IPTG was added into the medium, the level of Rrp2-FLAG produced in OY179 was ∼3-fold lower than the level of Rrp2 observed in WT strain 297. When bacteria were cultivated in BSK-II containing 30-µM IPTG, Rrp2-FLAG was produced at a level commensurate with the level of Rrp2 in WT strain 297. When 50- or 100-µM IPTG was added into the medium, Rrp2-FLAG produced in OY179 was 3.5-fold or 10.3-fold higher, respectively, than the level of Rrp2 in strain 297. In addition, there was a close correlation between the levels of Rrp2-FLAG and the levels of RpoS and OspC (Fig. 5A and 5B). Taken together, the increased synthesis of RpoS was coincident with the IPTG-inducible production of Rrp2-FLAG, supporting that Rrp2 activates the RpoN-RpoS pathway in *B. burgdorferi*.

### Rrp2 Indirectly Controls the Expression of *OspC* and *DbpA* via RpoS

**Figure 6:**
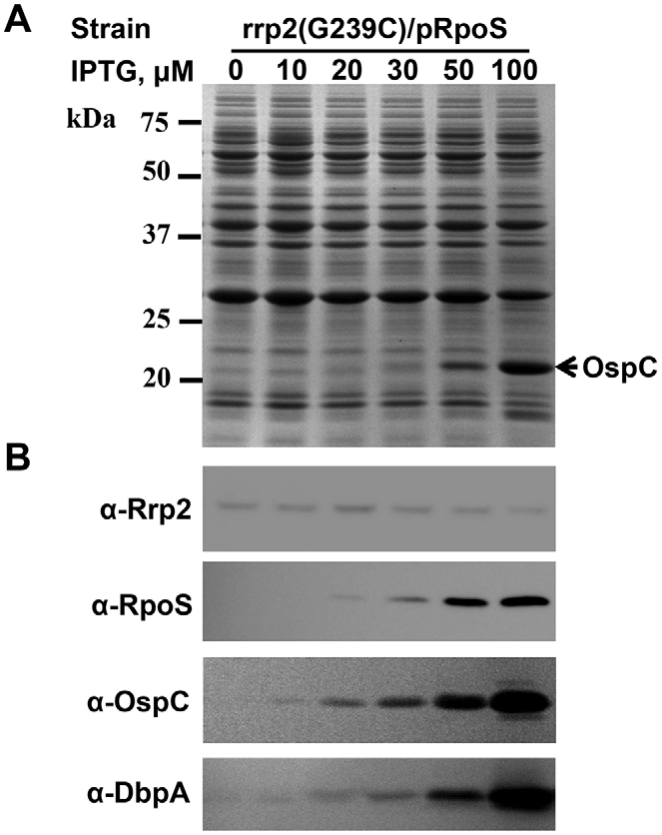
Rrp2 controls expression of *OspC* and *DbpA* via RpoS. (A, B) the *rrp2* point mutant *rrp2*(G239C) harboring the IPTG-inducible *rpoS* construct (pRpoS) was grown at 37°C with various concentrations of IPTG and gene expression was analyzed by SDS-PAGE (A) and immunoblot (B). The arrow in (A) indicates OspC. Specific antibodies, denoted as α-, used in the immunoblot (B) are indicated on the left.

Although the expression of *ospC* and *dbpA* was found to be lost in the *rrp2* point mutant [10], [29], [45], how Rrp2 ultimately controls the expression of these key lipoproteins has remained somewhat unclear. Given that (1) the expression of *ospC* and *dbpA* are directly regulated by RpoS through RpoS-specific promoters [74]–[76], and (2) *rpoS* transcription is abolished in the *rrp2* point mutant [10], [29], [45], we have hypothesized that Rrp2 likely regulates the expression of *rpoS* which, in turn, influences *ospC* and *dbpA* expression. To further test this hypothesis, we generated an IPTG-inducible *rpoS* expression shuttle construct pRpoS (i.e., pOY110) [30], in which *rpoS* expression is controlled solely by the IPTG-inducible PpQE30 promoter. This construct was introduced into the *rrp2* point mutant OY01, to investigate whether the IPTG-induced RpoS could restore *ospC* and *dbpA* expression in this mutant. As shown in Fig. 6A and 6B, when RpoS was induced from pRpoS by IPTG, production of OspC and DbpA was consequently rescued. As aforementioned, when the *rrp2* point mutation was complemented by the IPTG-inducible *rrp2* expression construct pRrp2, expression of *ospC* was restored in the complemented strain OY160 (Fig. 1B and 1C). Consistent with previous findings [69], however, when pRrp2 was introduced into a *B. burgdorferi rpoS* mutant AH206 (Δ*rpoS*), RpoS and OspC were not produced in this strain, despite the fact that Rrp2 synthesis was induced by IPTG (Fig. S2). These combined data suggest that controlled induction of RpoS can overcome the Rrp2 deficiency, which constitutes compelling evidence that Rrp2 indirectly controls *ospC* and *dbpA* expression via RpoS.

## Conclusions

This is the first study, to our knowledge, to show that the level of Rrp2 produced in *B. burgdorferi* directly correlates with RpoS levels. Moreover, our study provides further validation for the application of an IPTG-inducible expression system [65], [66] for assessing *B. burgdorferi* gene regulation. By using this system, an *rrp2* conditional lethal mutant was generated. Given the inability to inactivate *rrp2* via conventional deletion or insertion mutagenesis, and the fact that both σ^54^ and RpoS can be readily inactivated in *B. burgdorferi* [14], [18], [29], [48], [49], Rrp2 likely also controls the expression of genes independent of σ^54^ or RpoS control, which in turn suggests that Rrp2 may function as a unique bEBP (other characterized bEBPs activate only σ^54^–dependent promoters). Our new conditional mutant thus provides an innovative way not only to define the direct control of Rrp2 over the central RpoN-RpoS pathway, but also to interrogate the overall regulatory role of Rrp2 in *B. burgdorferi* gene regulation. For example, by profiling gene expression in this new conditional mutant, it may be possible to identify novel Rrp2-controlled, σ^54^- or RpoS-independent genes essential for *B. burgdorferi* growth or survival. Such studies will likely uncover new virulence-associated genes important for spirochetal pathogenesis, but also will provide the first definitive evidence that Rrp2 acts as an atypical bEBP to orchestrate virulence expression in *B. burgdorferi*. Although Rrp2 was presumed to be activated through phosphorylation [10], [29], [45], it still remains unanswered whether or how phosphorylated Rrp2 dynamically controls *rpoS* expression. Future work, such as pulse-chase analyses of the *rrp2* point mutant OY01 *trans*-complemented with *rrp2* variants, may help address these questions.

## Supporting Information

Figure S1 G**rowth of the** ***rrp2*** **conditional mutant OY179** ***in vitro*** **.** *B. burgdorferi* was inoculated into BSK-II medium with various concentrations of IPTG at 1000 spirochetes/ml. Spirochetes were enumerated using darkfield microscopy. Values are the means from three independent experiments. Error bars indicate standard deviations (*n* = 3).
(TIF)

Figure S2 **Overexpression of Rrp2 does not restore expression of** ***OspC*** **and** ***DbpA*** **in the** ***RpoS*** **mutant.** The *rpoS* point mutant AH206 harboring the IPTG-inducible *rrp2* construct (pRrp2) was grown at 37°C with various concentrations of IPTG and gene expression was analyzed by SDS-PAGE (A) and immunoblot (B). RpoS and OspC were not detected in this strain, which is consistent with previous findings [69]. Specific antibodies, denoted as α-, used in the immunoblot (B) are indicated on the left.
(TIF)

Table S1 **Oligonucleotide primers used in this study.**
(DOCX)
