# Supplementary figures and images for: Synthesis of RpoS Is Dependent on a Putative Enhancer Binding Protein Rrp2 in Borrelia burgdorferi
Source: PLoS One. 2014 May 8;9(5):e96917. doi: 10.1371/journal.pone.0096917 (PMC4014564; doi:10.1371/journal.pone.0096917)

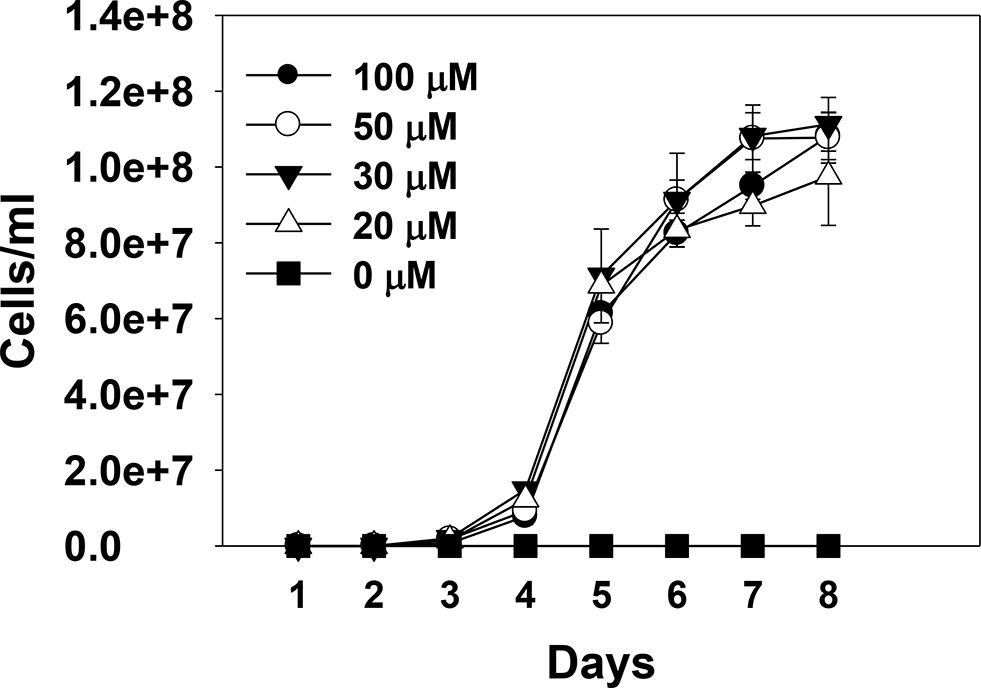

Supplement: Figure S1 — Growth of the rrp2 conditional mutant OY179 in vitro . B. burgdorferi was inoculated into BSK-II medium with various concentrations of IPTG at 1000 spirochetes/ml. Spirochetes were enumerated using darkfield microscopy. Values are the means from three independent experiments. Error bars indicate standard deviations (n = 3). (TIF) [file pone.0096917.s001.tif]

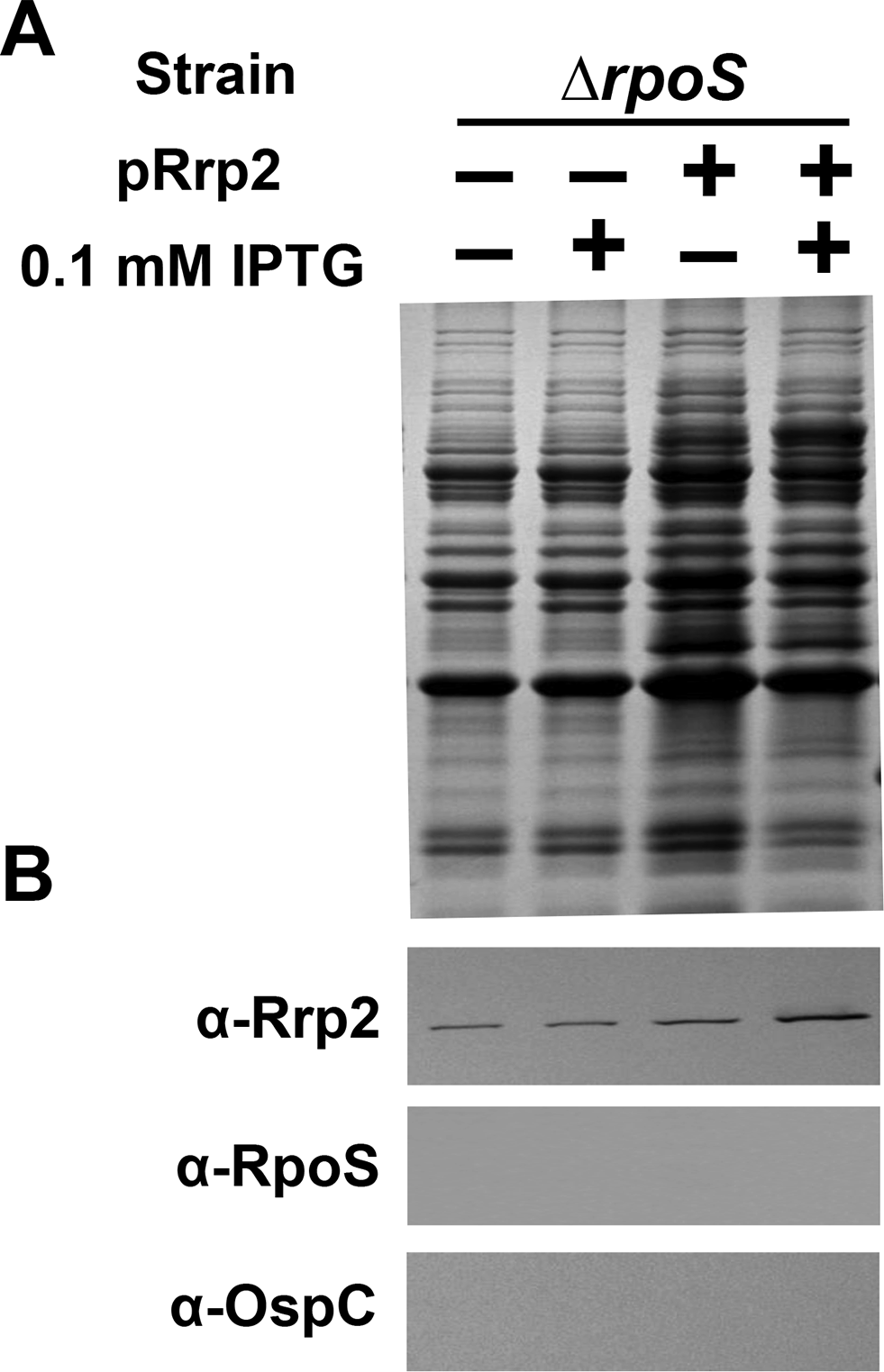

Supplement: Figure S2 — Overexpression of Rrp2 does not restore expression of OspC and DbpA in the RpoS mutant. The rpoS point mutant AH206 harboring the IPTG-inducible rrp2 construct (pRrp2) was grown at 37°C with various concentrations of IPTG and gene expression was analyzed by SDS-PAGE (A) and immunoblot (B). RpoS and OspC were not detected in this strain, which is consistent with previous findings [69]. Specific antibodies, denoted as α-, used in the immunoblot (B) are indicated on the left. (TIF) [file pone.0096917.s002.tif]
