# Supplementary material for: Synthesis of RpoS Is Dependent on a Putative Enhancer Binding Protein Rrp2 in Borrelia burgdorferi
Source: PLoS One. 2014 May 8;9(5):e96917. doi: 10.1371/journal.pone.0096917 (PMC4014564; doi:10.1371/journal.pone.0096917)
Supplement: Table S1 — Oligonucleotide primers used in this study. (DOCX) [file pone.0096917.s003.docx]

**Table S1. Oligonucleotide primers used in this study.**

| **PCR primer** |  | |  |
| --- | --- | --- | --- |
| **primer** | **Sequence, 5'-3'** | |  |
| 009F | GTTTGTTGCAGCCAGAAGCCTGAT | | |
| 009R | TCTAACAGCCCACGGAACAGTTGA | | |
| 68F | TATGCCTCTTCCGACCATCAAGCA | | |
| 68R | AGGCAGTTCCATAGGATGGCAAGA | | |
| 86F | GGCGCTCTCAAGGCAAGATCCTGTT | | |
| 86R | TAATGGCGCGCCAGCAAATGCGCCGAAATACT | | |
| 87F | TAATGGCGCGCCTGGCATCTACAGCAGAATCA | | |
| 87R | GCGCAGTTCCAATCTCAAACGGCA | | |
| 112F | TAAGGCTTGATGAAACAACGCGGC | | |
| 112R | CGTCGTGCACAACAATGGTGACTT | | |
| 235F | TAATGGCGCGCCCAAAGAGGGCAAAGGCACTA | | |
| 235R | GGGCCCTTGTTTGTTGCAGCCAGAAG | | |
| 303F | GAGAAATTACATATGAGCAAAATACTTGTAGCTGAT | | |
| 303R | TATCATATGTTAATCAATATTATATTCGATT | | |
| 263R | TATCATATGTCACTTATCGTCGTCATCCTTGTAATCATCAATATTATATTCGATAA | | |
|  |  | |  |
| **qPCR primers** | | |  |
| **gene** | | **Forward (5'-3')** | **Reverse (5'-3')** |
| *flaB* | | TGATTAGCCTGCGCAATCATT | AATGACAGATGAGGTTGTAGCAGC |
| *rrp2* | | ACAGCCCACGGAACAGTTG | AAAAAATCATAAGCACCCTCTCTCA |
| *rpoS* | | CTGGACAAAGAAATAGAGGGATCTG | CAAGGGTAATTTCAGGGTTAAAAGAA |
